# A nystagmus extraction system using artificial intelligence for video-nystagmography

**DOI:** 10.1038/s41598-023-39104-7

**Published:** 2023-07-24

**Authors:** Yerin Lee, Sena Lee, Junghun Han, Young Joon Seo, Sejung Yang

**Affiliations:** 1 Department of Biomedical Engineering, Yonsei University, Wonju, 26493 Republic of Korea; 2 Department of Precision Medicine, Yonsei University Wonju College of Medicine, Wonju, 26426 Republic of Korea; 3 Research Institute of Hearing Enhancement, Yonsei University Wonju College of Medicine, Wonju, 26426 Republic of Korea; 4 Department of Otorhinolaryngology, Yonsei University Wonju College of Medicine, Wonju, 26426 Republic of Korea

**Keywords:** Computational biology and bioinformatics, Peripheral nervous system, Diagnosis

## Abstract

Benign paroxysmal positional vertigo (BPPV), the most common vestibular disorder, is diagnosed by an examiner changing the posture of the examinee and inducing nystagmus. Among the diagnostic methods used to observe nystagmus, video-nystagmography has been widely used recently because it is non-invasive. A specialist with professional knowledge and training in vertigo diagnosis is needed to diagnose BPPV accurately, but the ratio of vertigo patients to specialists is too high, thus necessitating the need for automated diagnosis of BPPV. In this paper, a convolutional neural network-based nystagmus extraction system, ANyEye, optimized for video-nystagmography data is proposed. A pupil was segmented to track the exact pupil trajectory from real-world data obtained during field inspection. A deep convolutional neural network model was trained with the new video-nystagmography dataset for the pupil segmentation task, and a compensation algorithm was designed to correct pupil position. In addition, a slippage detection algorithm based on moving averages was designed to eliminate the motion artifacts induced by goggle slippage. ANyEye outperformed other eye-tracking methods including learning and non-learning-based algorithms with five-pixel error detection rate of 91.26%.

## Introduction

Dizziness and vertigo are common symptoms, affecting approximately 20–30% of the population^1,2^. Vertigo, specifically, refers to the perception of motion when no actual motion is occurring relative to the Earth’s gravity^3^. Some experts in the field of dizziness differentiate vertigo as a symptom caused by disorders of the vestibular system, as opposed to general dizziness^4^. One prominent vestibular disorder is known as Benign Paroxysmal Positional Vertigo (BPPV), which accounts for a significant proportion of vertigo cases, diagnosed in approximately 17–47% of patients^5^. BPPV is characterized by episodes of vertigo that are triggered by specific head movements, such as turning in bed, bending over, or looking up^6^. The underlying cause of BPPV is the displacement of tiny calcium carbonate crystals, known as otoliths, which are responsible for sensing gravity in the utricles and entering the semicircular canals. This displacement can occur due to various factors, including illness or the natural aging process. BPPV can be further classified into two subtypes: cannalithiasis and cupulithiasis. In cannalithiasis, the otoliths freely move within the semicircular canals, while in cupulithiasis, the otoliths adhere to the cupula, although the latter subtype is less common^7,8^. Depending on the affected semicircular canal, BPPV is categorized as posterior canal BPPV, lateral canal BPPV, or anterior canal BPPV, with posterior canal BPPV being the most frequently observed subtype^9^.

BPPV is a multifactorial disease characterized by the displacement of otoliths, which are responsible for detecting gravity in the utricles and entering the semicircular canals. While aging and certain illnesses are commonly linked to BPPV, it can arise from various etiologies. Aging is a significant contributor, accounting for approximately 40% of BPPV cases^4^. Additionally, traumatic events, such as head injuries or accidents, have been associated with BPPV, contributing to around 20% of cases. Inflammation within the inner ear, resulting from conditions like vestibular neuritis or labyrinthitis, can also trigger BPPV in approximately 10–15% of individuals. Moreover, certain medical conditions and comorbidities have been implicated as potential risk factors for BPPV. For instance, vitamin D deficiency has been identified as a possible contributing factor, with studies suggesting an association in about 25% of BPPV cases^5^. Other underlying factors, including Ménière’s disease, vestibular migraine, or autoimmune disorders, may contribute to a smaller percentage of BPPV cases. It’s important to note that while these percentages provide a general overview, the exact contribution of each etiology can vary among individuals, and BPPV can often arise from a combination of factors. Understanding the diverse etiologies of BPPV is crucial for effective diagnosis, management, and treatment strategies.

BPPV is diagnosed by an examiner changing the posture of the examinee, which causes the vestibulo-ocular reflex. The method of diagnosis depends on the location of misplaced otoliths. Posterior canal BPPV is diagnosed using the Dix–Hallpike maneuver, which was first introduced in 1952^10^. According to this maneuver, the examinee initially sits on the bed, and then the examiner turns the head of the examinee 45 degrees to the left or right and makes the examinee quickly lay down to cause dizziness. Torsional nystagmus occurs in patients with positive results when the head is lowered. Lateral canal BPPV is diagnosed using the supine roll maneuver^11^. If the subject is in the supine position and the head of the examinee is turned quickly, horizontal nystagmus occurs in positive patients.

Nystagmus is an involuntary periodic eye movement characterized by a slow phase moving slowly in one direction and a fast phase that quickly returns to its original position^12^. BPPV nystagmus is caused by improper stimulation of semicircular canal receptor hair cells according to changes in the head position^13^. Nystagmus occurs in various directions depending on the location of the misplaced otoliths and includes horizontal nystagmus, vertical nystagmus, and torsional nystagmus. The diagnostic methods used to observe nystagmus include electro-oculography, video-nystagmography, and scleral search coil technology^14,15^. Among them, video-nystagmography utilizing an infrared camera has been widely used in recent years because it is non-invasive and does not cause pain to the examinee^16^. However, video-nystagmography has limitations, such as lack of dimension, goggle slippages, and manual evaluation of data^17^. Therefore, only a specialist with professional knowledge and training in vertigo can diagnose BPPV accurately using video-nystagmography data obtained through the positional tests. However, the number of specialists is insufficient to accommodate all vertigo patients, thus necessitating automated diagnosis of video-nystagmography data.

Owing to recent developments in image processing technology and machine learning, several studies have attempted to detect and diagnose nystagmus using video-nystagmography images. Lim et al.^18^ extracted the pupil trajectory and iris pattern from a video in which 10 types of tests were performed and obtained an amplitude in three directions to distinguish eight types of BPPV with a deep learning model. Slama et al.^19^ extracted pupil trajectories from caloric and kinetic test videos and extracted various features to diagnose vestibular neuritis using support vector machines. Reinhardt et al.^20^ developed an algorithm that detects the eye using a cascade classifier in a webcam image to obtain eye trajectories and determine the time when nystagmus occurs. Zhang et al.^21^ used a two-stage deep learning model that selected invalid frames of video nystagmography videos and found that torsional nystagmus occurred. However, most of these studies used the Hough transform or machine learning to find the trajectory of nystagmus, with deep learning only used for diagnosis. Considering the amplitude, speed, and direction of nystagmus are important in BPPV diagnosis, these methods have limitations because it is necessary to accurately track the waveform of nystagmus to measure its characteristics.

Pupil detection for eye tracking and gaze estimation has been studied over the last few years, with growing attention focused on commercial eye trackers. A histogram-based algorithm was developed^22,23^. For algorithms such as Starburst^24^, ExCuSe^25^, ElSe^26^, and PuRe^27^, ellipse fitting was applied after detecting the edge of the pupil. However, these algorithms have not enabled smooth detection in noisy environments. With the development of convolutional neural networks, deep learning applications in the field of computer vision have been actively conducted recently. Accordingly, eye-tracking algorithms that apply neural networks have emerged, such as PupilNet^28^, DeepEye^29^, and DeepVOG^30^. All these attempts have focused on finding pupil location or dividing the pupil itself using convolutional neural networks (CNN). EllSeg^31^ achieved a higher performance than previous algorithms by splitting the pupil and iris regions simultaneously with a CNN and approximating ellipses via representation maps. However, the dataset used for learning is limited to commercially available eye-tracker images. In addition, considering the dataset overfitting characteristics of CNN, the images of eye trackers and video-nystagmography have significantly different environments and conditions, making them incompatible with each other. Therefore, it is necessary to train a CNN-based tracking algorithm using video nystagmography data.

When performing video-nystagmography, slippage occurs because of the heavy weight of the device or quick changes in posture when performing the diagnosis^32–34^, causing motion artifacts in the pupil trajectory. As device slippage occurs at a high frequency, significant efforts have been made to prevent slipping in the diagnostic stage^35,36^. According to our research, studies on removing motion artifacts from the perspective of signal processing have not been conducted.

**Figure 1:**
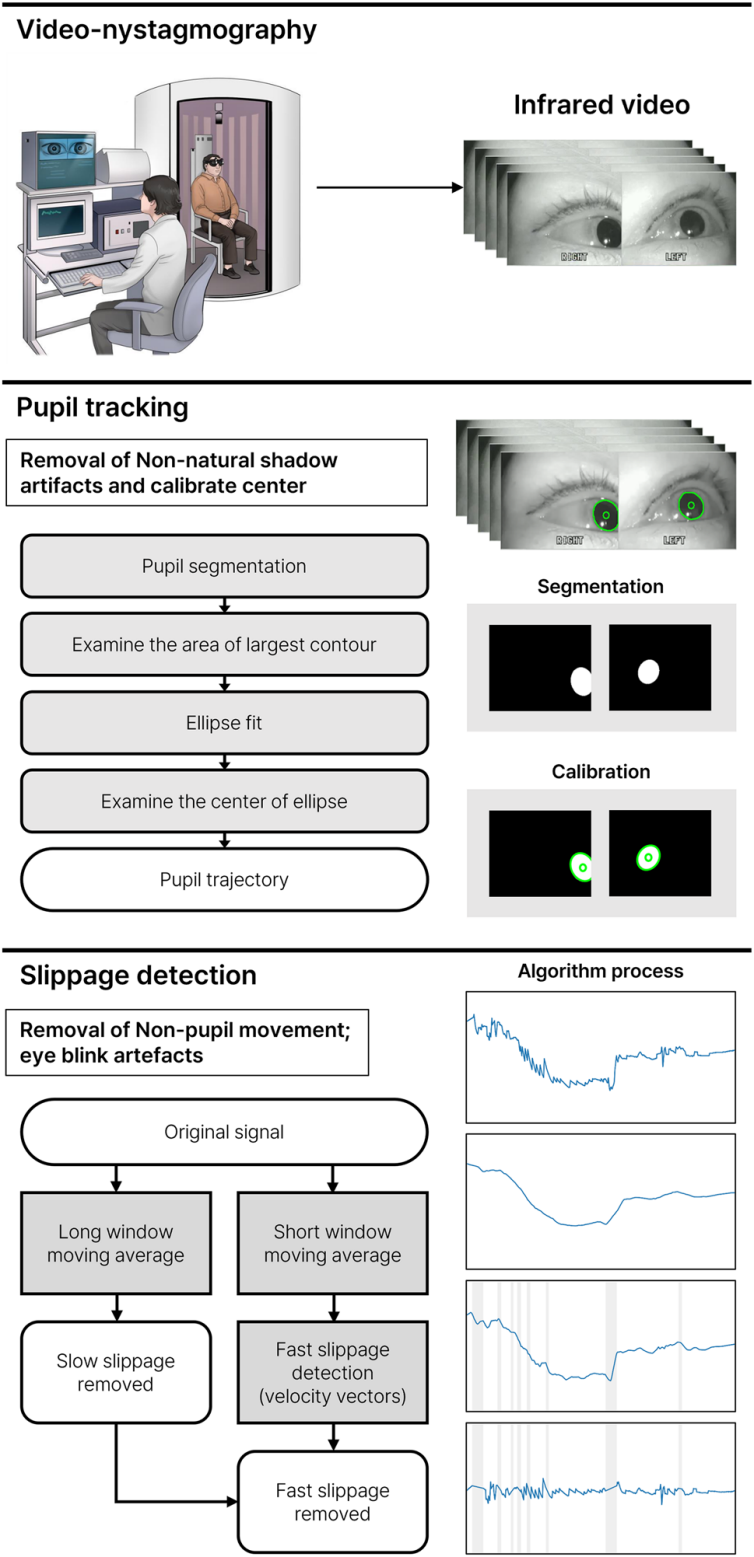
Overview of ANyEye framework.

In this paper, a deep learning-based nystagmus extraction system optimized for video-nystagmography (ANyEye) is proposed in the first study for automating BPPV diagnosis. ANyEye consists of two parts: eye-tracking and slippage detection (Fig. 1). In eye-tracking process, the pupil is segmented through CNN model to track the exact pupil trajectory from noisy real-world data obtained during diagnosis, and a compensation algorithm corrects the position to obtain more precise center. This process is optimized to dark-field video-nystagmography data which has different environment from typical open-space commercial eye tracker data. In addition, a slippage detection algorithm was designed based on two-stage moving average to remove motion artifacts caused by the slippage of video-nystagmography devices.

## Methods

### Data acquisition

#### Data resource

This study was approved by the Institutional Ethics Committee of Yonsei University Wonju College of Medicine, Wonju, Korea (No. CR319082), and informed consent was obtained from all subjects. All methods were performed in accordance with the principles of the Declaration of Helsinki. The dataset used in the experiment comprised video-nystagmography infrared videos of 46 posterior semicircular canal BPPV patients and nine lateral semicircular canal BPPV patients, acquired retrospectively at Yonsei University Wonju Severance Hospital. The video-nystagmography goggles (Easy-Eyes, SLMED, Seoul, Korea) weighed 330 g with a resolution of 640 × 480 px and supported 30 frames per second. The screen consists of a section with infrared camera images showing the right and left eyes and a section with images showing the overall appearance of the examination environment. In one patient video, the results of the spontaneous nystagmus test, Dix–Hallpike test, supine roll test, head-shaking test, and bow-and-lean test were recorded.

#### Positional test dataset

Two datasets were used for the experiments. The first was a dataset with labels of positional tests and video-nystagmography videos conducted on 48 posterior semicircular canal BPPV patients and four lateral semicircular canal BPPV patients. A total of 66 tests were conducted, including several tests conducted on the same patient. Approximately 10 s of the section to check for nystagmus after each test were manually edited and indicated by otolaryngologists, with 165 video clips obtained from the original data.

**Figure 2:**
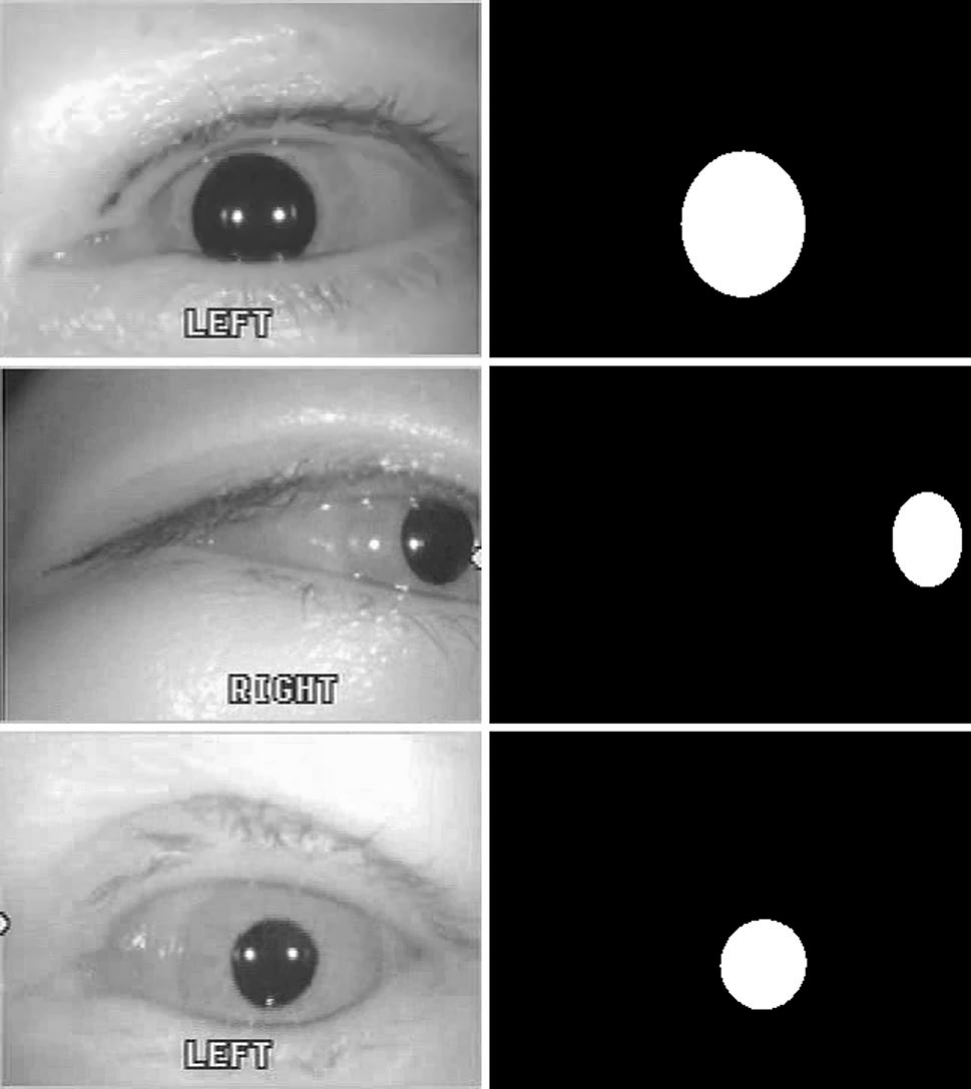
Examples of acquired dataset.

The ground truths of the pupil region for the deep learning model were generated by four researchers using a self-constructed labeling application. Participants were required to specify the boundary of the pupil with at least ten points to generate ground truths. The data points were then used to approximate the ellipse using the RANSAC algorithm^37^, with an approximated ellipse displayed on the screen for confirmation. If the user determines that the displayed ellipse matches the pupil, the program automatically generates an image that designates the inside of the ellipse as the pupil. The frame was selected using two rules: (1) 15 frames from the first frame and (2) one frame with an interval of 20 frames. A total of 8284 frames were obtained, with the data divided in a ratio of 7:1:2 for use as training, validation, and test sets for cross-validation (Table 1). Examples of the data and ground truths are shown in Fig. 2.

**Table 1:** Dataset for cross validation.

	Videos	Images
Train	115	5802
Validation	16	848
Test	34	1784
Total	165	8434

#### Slippage dataset

The data used for evaluating the slippage detection algorithm comprised eight video infrared videos. The test was performed by an otolaryngologist on eight patients with lateral semicircular BPPV. All patients showed geotropic nystagmus, seven of the eight subjects showed left-direction nystagmus, and one subject showed right-direction nystagmus. The acquired data were observed to analyze slippage-induced motion artifacts, with the section where the slippage occurred indicated by two otolaryngologists.

### Pupil segmentation

The proposed algorithm ANyEye first recognizes pupil by a CNN model-based approach. The backbone model was trained with positional test dataset to adapt the features of the video-nystagmography data. All inputs were resized to 256 × 256 pixels and normalized to a mean of 0.5 with a standard deviation of 0.5. In the training phase, random rotation with a range of − 10° to 10°, random scaling with a ratio of 0.8–1.2, and random shift with a ratio of 0–0.5 were applied to the input image. The total loss ${L}_{total}$ was calculated by adding the binary cross-entropy loss ${L}_{BCE}$ and dice loss ${L}_{dice}$ using the following equations:

1 $$L_{BCE} = - \frac{1}{N}\sum\nolimits_{i = 1}^{N} {} \left( {y_{i} \cdot \log x_{i} + \left( {1 - y_{i} } \right) \cdot \log \left( {1 - x_{i} } \right)} \right)$$

2 $$L_{dice} = 2 \times \frac{{\left| {X \cap Y} \right|}}{\left| X \right| + \left| Y \right|}$$

3 $$L_{total} = L_{BCE} + L_{dice}$$

where $X$ is the input data, $Y$ is the output, and ${x}_{i}\in X,{y}_{i}\in Y, i=1, \dots , N$. The Adam optimizer^38^ was used with a learning rate of 0.001 over 500 epochs using the early stopping method with patience of 100. The best model was selected based on the loss of the validation set. A batch size of 16 was selected, considering our GPU, NVIDIA GeForce RTX 3090. The Pytorch framework^39^ was used for implementing the CNN models in Python 3.6.8.

### Compensation algorithm

**Figure 3:**
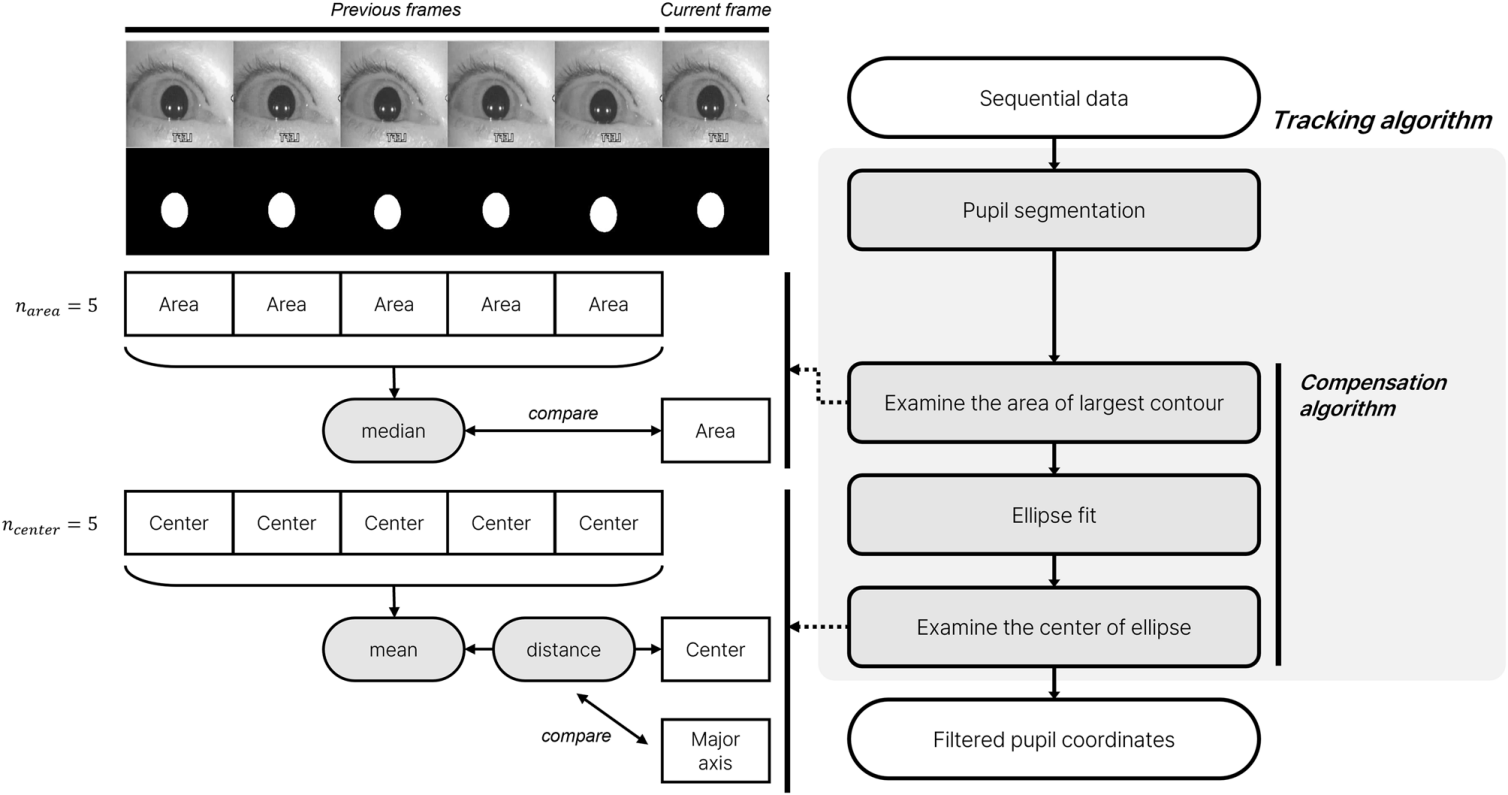
Process followed by tracking algorithm.

In the output of the segmentation model, the pupils may not be fully estimated; therefore, additional steps are required. As the pupils were elliptical and had similar sizes throughout one video, we designed an algorithm based on this idea (Fig. 3). The algorithm first compares the area of the estimated pupil region with the segmentation method with the previous *i* frames and determines whether the size of the selected region is within a certain range. If the area of the region is similar to that in the previous frames, the ellipse is approximated from the selected region^40^, with the ellipse center defined as the pupil location. The brightness of the shadow on the side of the data is similar to that of the pupil, which may confuse the segmentation models. The algorithm stores the positions and compares the distance of movement from the previous frame with the major axis of the ellipse from the previous *j* frames to prevent misjudgment. If the distance is abnormally long, the frame is indicated as invalid and removed from the data. The pupil coordinates of the frames removed by the algorithm are estimated using linear interpolation. We empirically set *i* and *j* as 5.

### Slippage detection

**Figure 4:**
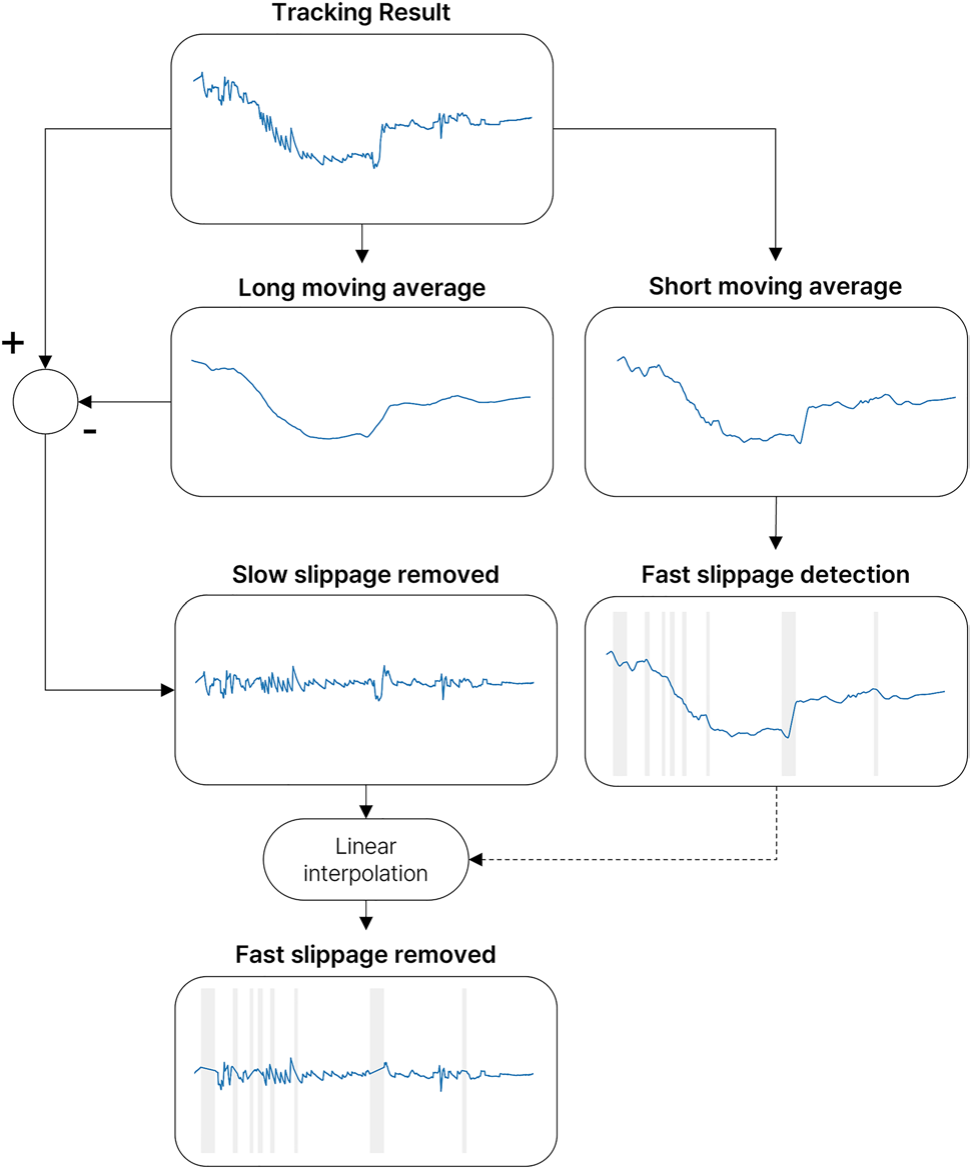
Process followed by slippage detection algorithm.

Previous nystagmus detection algorithms based on conventional signal processing methods had difficulties separating motion artifacts from nystagmus patterns, resulting in significant errors in the detection of nystagmus. In ANyEye, an algorithm was additionally constructed with a moving average to eliminate motion artifacts caused by the movement of goggles or eyes. Two types of slippages occur in eye-movement signals: fast slippage and slow slippage. The fast slippage tends to not include nystagmus because the position changes quickly in a short time, and the slow slippages often included nystagmus because the position changes slowly over a long period of time. The causes of the noise included voluntary causes such as movement due to the weight of the device and change of gaze of the subject, and involuntary causes such as the examiner adjusting the position or the camera of the device. Among them, the noise induced by the position of the eye changes in the screen was defined as slippage. Slow slippage was mainly caused by sliding due to the weight of the device when the subject was still, and fast slippage was mainly caused by shaking because the device was not fixed to the head of the subject during rapid posture change during the positional test. The algorithm generates two moving average values from the original signal, i.e., the short- and long-window moving averages, to remove these two types of slippages using the following equations:

4 $$MA(n) = 1/(w + 1)\sum\nolimits_{(m = n - w/2)}^{(n + w/2)} {x(m)} ,w = window \cdot fps$$

where $MA(n)$ is the moving average signal, $n$ is the nth frame, $x(n)$ is the eye movement signal, $window$ is the window length in seconds, and $fps$ is the frames per second of the video. Slow slippage was removed by subtracting the moving average of the long window from the original signal. Next, fast slippage was detected using a short-window moving average signal. After calculating the short-window moving averages of the x- and y-axis data, the vector velocity was calculated using the following equation:

5 $$v_{total} = \sqrt {v_{x}^{2} + v_{y}^{2} }$$

where ${v}_{x}$ is the velocity of the x-axis, and ${v}_{y}$ is the velocity of the y-axis. The threshold was used to find the section that moved faster than a specific speed. When the interval among the selected sections was 0.7 s or less, it was determined that the speed was instantaneously slowed during the slippage; when the interval was 0.3 s or less, it was determined that the interval was not a slippage and excluded. The fast slippage sections were removed from the slow-slippage-removed signal using linear interpolation. A flowchart of the slippage detection algorithm is shown in Fig. 4.

## Results

### Segmentation performance

The test set outputs of CNN models were compared to evaluate the segmentation performance. The backbone model was trained with positional test dataset to adapt the features of the video-nystagmography data. The architectures and specific configurations for the CNN network used in this paper are detailed in the “Supplementary Materials” (Figure S1, Table S1, Table S2). Using the early stopping rule, U-Net model with 95 epochs, U-Net++ L2 model with 217 epochs, U-Net++ L3 model with 231 epochs, and U-Net++ L4 model with 269 epochs were chosen as the best models. The performances of the four pupil segmentation methods were evaluated using five metrics based on the ground truths, and the mean of inference time for one batch was measured (Table 2). The Dice coefficient was used to calculate the similarity between the two samples. The area under the receiver operating characteristic curve is an indicator of the performance of a classifier. A curve drawn on a graph with the false positive rate and true positive rate as the axes is called the receiver operating characteristic (ROC) curve, and the area below the ROC curve is called the area under the ROC curve (AUROC).

**Table 2:** Performance comparison of segmentation methods. The value highlighted in bold represents the best performance achieved.

Methods	Accuracy	Precision	Recall	Dice	AUROC	Inference time* (milliseconds)
UNet	0.99	0.95	0.95	0.93	**0.990**	91.49
UNet++ L2	0.99	0.95	0.94	0.93	0.989	74.30
UNet++ L3	0.99	0.95	0.96	0.94	0.986	84.00
UNet++ L4	0.99	0.95	0.96	0.94	0.987	102.66

*Mean.

### Tracking evaluations

The detection rates of the conventional methods and the proposed method were compared with the results of 34 test set videos to evaluate the tracking performance. The detection error ${\mathcal{E}}_{d}$ was defined as L2 norm between the estimated center of the pupil ${p}_{i}$ and the ground truth ${q}_{i}$ to evaluate the performance of the eye-tracking algorithm.

6 $${\mathcal{E}}_{d} \left( {p_{i} , q_{i} } \right) = \left\| {p_{i} - q_{i} } \right\|_{2}$$

The detection rate, which is the ratio of the number of samples with a detection error ${\mathcal{E}}_{d}$ less than a specific threshold value ${t}_{d}$ to the total number of samples, was calculated. Typically, the detection rate is compared when the ${t}_{d}$ is 5 pixels; thus, the performance between algorithms is compared based on the detection rate at a 5-pixel error.

**Figure 5:**
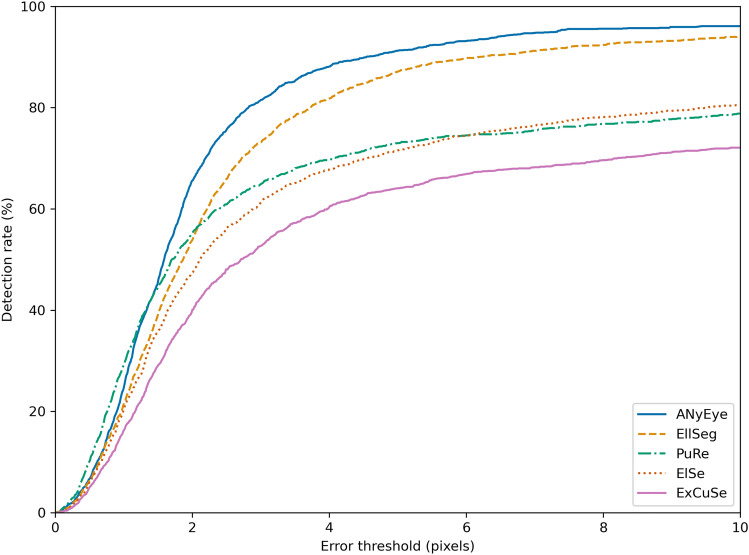
Comparison of detection rates of EllSeg, PuRe, ElSe, ExCuSe, and ANyEye.

**Figure 6:**
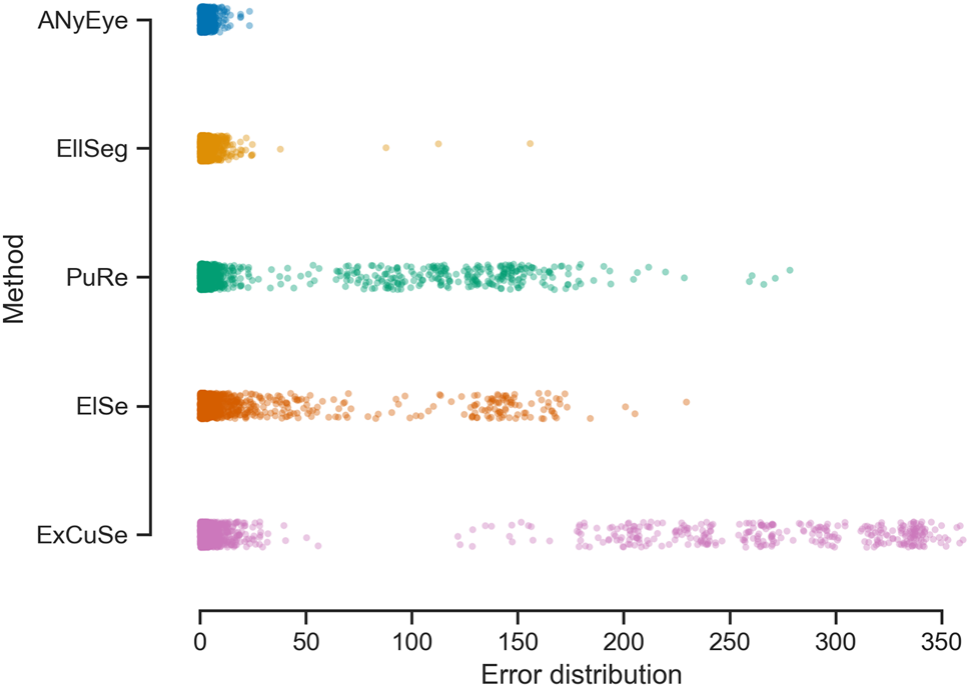
Strip plot of detection errors in test set using EllSeg, PuRe, ElSe, ExCuSe, and ANyEye.

**Figure 7:**
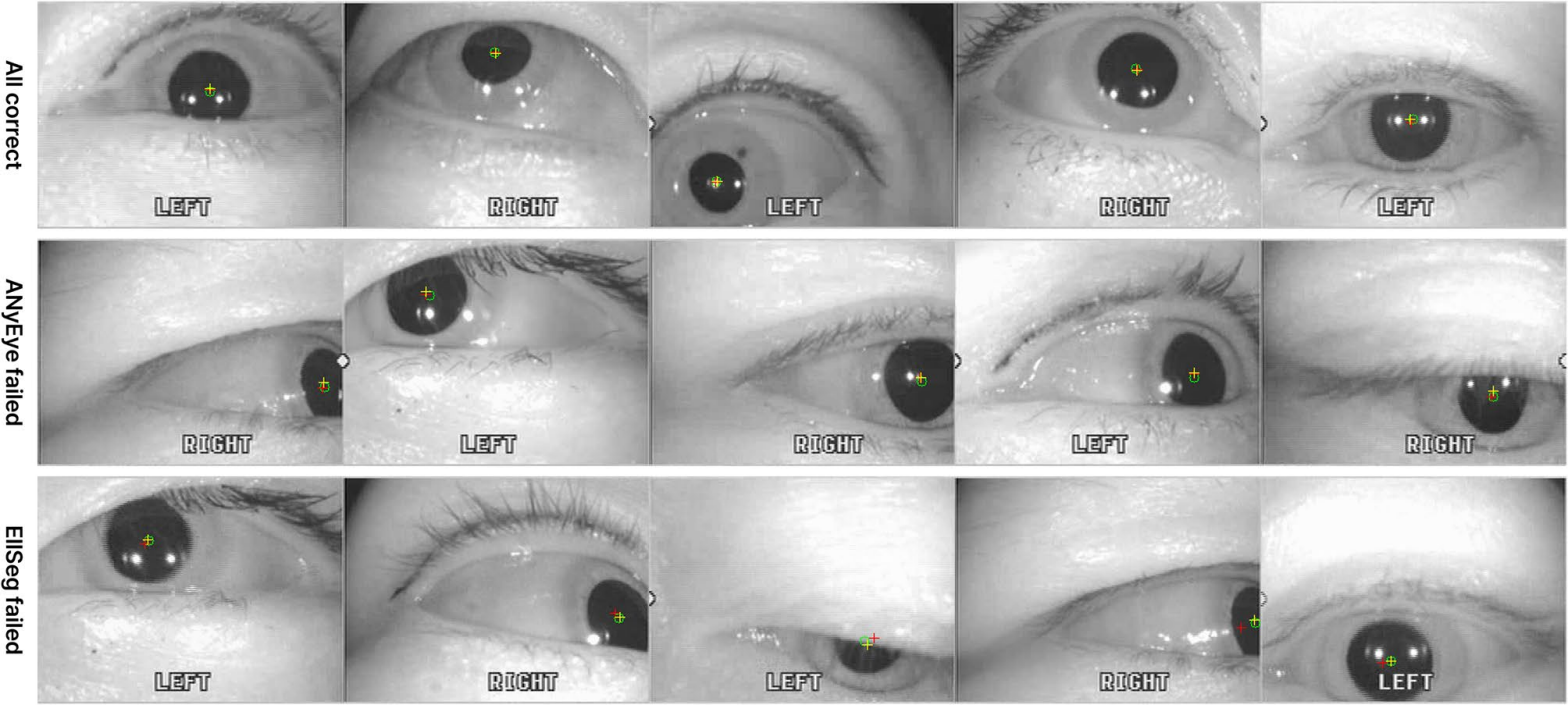
Examples of tracking algorithm result. Green circle indicates the ground truth of the input with 5-px radius. Result of EllSeg and ANyEye are cross marked with red and yellow, respectively.

Figure 5 shows the detection rates of ANyEye, EllSeg, PuRe, ElSe, and ExCuSe by adjusting the error threshold ${t}_{d}$ from 0 to 10. The statistics of detection errors and detection rates up to an error of five pixels for AnyEye, EllSeg, PuRe, ElSe, and ExCuSe are shown on Table 3. ANyEye scored the highest detection rate compared to previous methods with five-pixel error detection rate of 91.26%. Figure 6 shows the distribution of detection errors to the result of five eye-tracking methods including ANyEye. Figure 7 shows the tracking results marked on the examples from the test set. The sample input and output of the ANyEye framework are provided in Figure S2 in the "Supplementary Materials," accompanied by a link to the Python code for evaluation.

**Table 3:** Detection error analysis of eye-tracking methods with test set videos.

Methods	Detection error* (pixels)	Detection rate at 5-pixel error (%)
ANyEye	2.05 ± 2.01 (0.02–23.44)	**91.26**
EllSeg	2.81 ± 5.76 (0.01–155.85)	87.11
PuRe	21.50 ± 47.10 (0.02–278.45)	72.98
ElSe	13.20 ± 33.88 (0.05–229.61)	71.52
ExCuSe	55.31 ± 108.73 (0.05–360.20)	64.07

The value highlighted in bold represents the best performance achieved.

*Mean ± standard deviation (minimum–maximum).

### Slippage detection results

**Figure 8:**
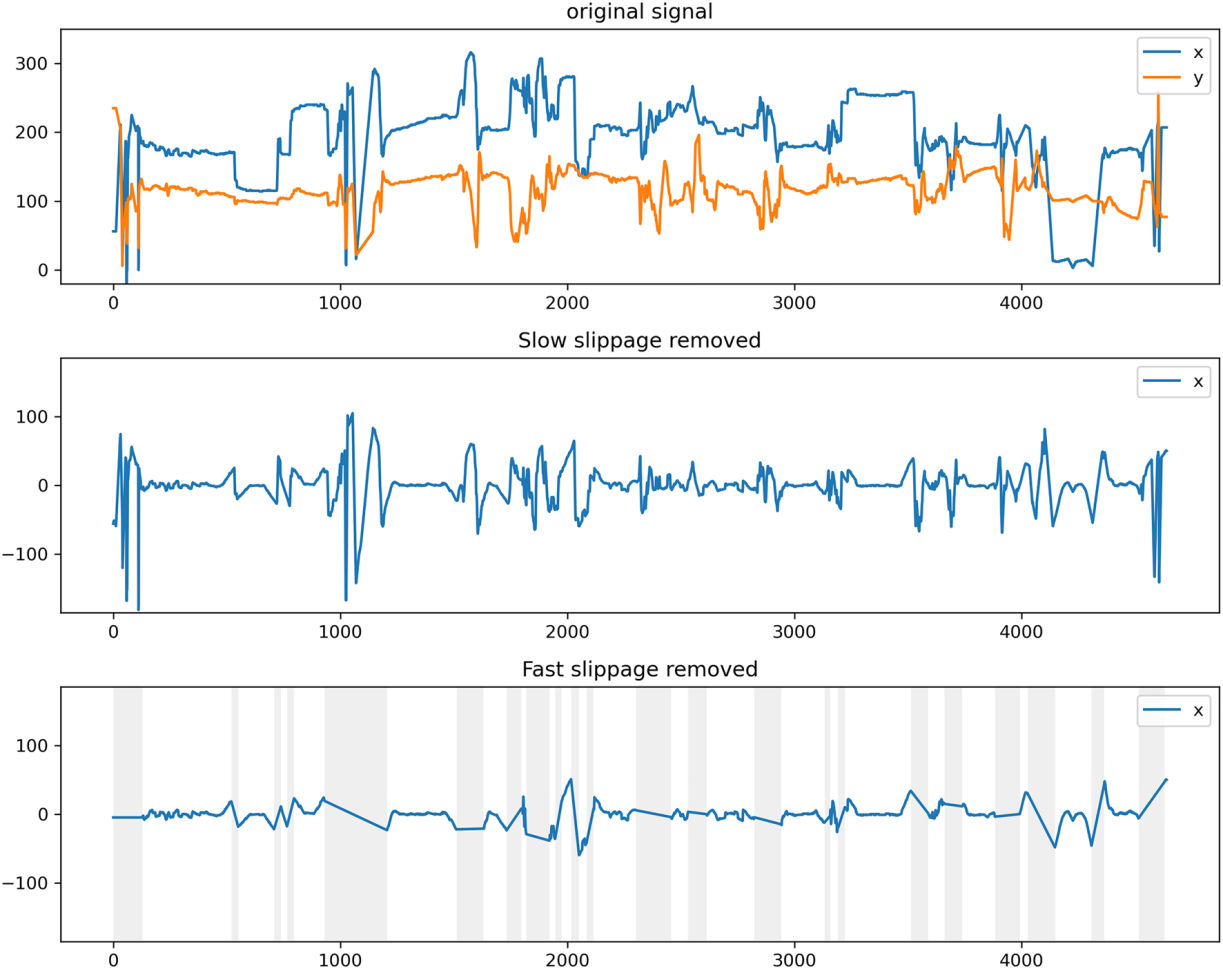
Example of slippage detection algorithm result. The gray region indicates detected fast slippage by the slippage detection algorithm.

**Figure 9:**
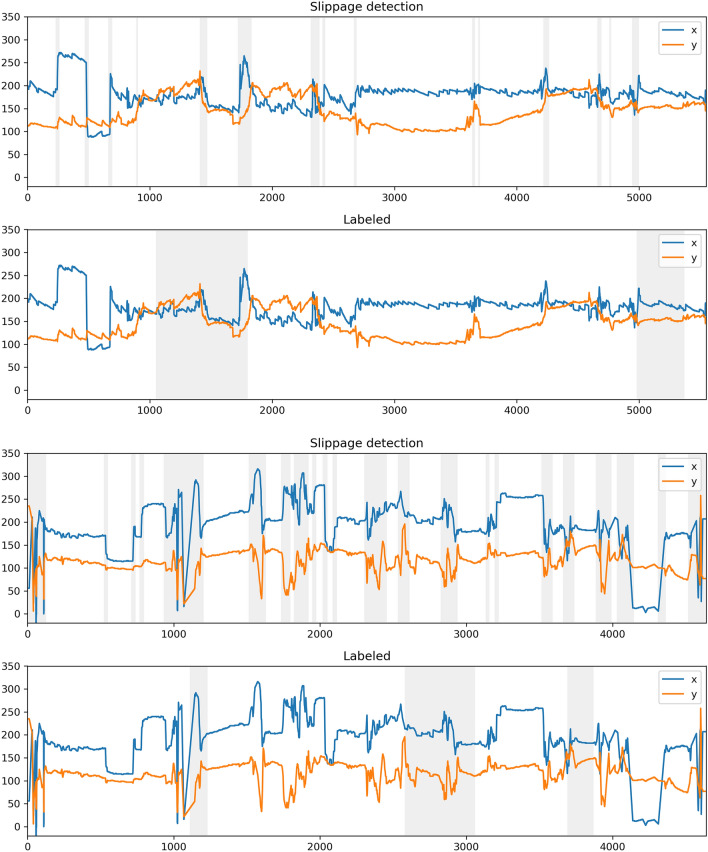
Comparison between detected fast slippages and labeled slippages. The gray region in the slippage detection plot indicates detected fast slippage by the slippage detection algorithm. The gray region in the labeled plot indicates slippages labeled by the experts.

The parameters of the slippage detection algorithm were selected empirically as 4 s for the long window and 1 s for the short window. Algorithm performance was verified by comparing the pupil trajectory signal and the results of the slippage detection algorithm (Fig. 8). The nystagmus waveform was maintained while the motion artifact due to gaze change or slippage was removed from the algorithm results. Next, the fast slippage detected by the slippage detection algorithm was compared with the slippage indicated by the expert (Fig. 9).

## Discussion

Nystagmus in BPPV can be observed in infrared video-nystagmography images. However, diagnosing BPPV requires expert knowledge as it is difficult for untrained non-experts to diagnose because nystagmus appears within a short range and in a short period. In the future, deep-learning nystagmus analysis or automated nystagmus analysis programs are expected to be developed to overcome the shortage of specialists and the rapidly increasing number of patients with dizziness. To accurately analyze the nystagmus, it is necessary to find the center of the pupil accurately. However, several factors interfere with finding the center of the pupil. If it is difficult to distinguish the pupil due to eyelashes or dark makeup, or if the center of the pupil changes due to slippage of the device, it may be difficult to automatically classify the nystagmus pattern appear in video-nystagmography data. In this study, a CNN was introduced to segment the pupil to obtain the exact eye trajectory, with CNN shortcomings addressed through a compensation algorithm. In addition, a moving-average-based slippage detection algorithm was developed to remove motion artifacts caused by frequent slippage of the device during diagnosis.

The algorithm proposed in this paper, ANyEye, first estimates the shape of the pupil from video nystagmography videos using CNN-based segmentation model. Four segmentation methods were tested: U-Net, UNet++ L2, UNet++ L3, and UNet++ L4. The U-Net architecture was selected based on AUROC among five metrics because robustness in the pupil segmentation process was the most important feature for eye tracking.

A compensation algorithm was designed to determine the exact center of the pupil from the region estimated using segmentation model. After selecting the connected component with the largest area in the segmentation result, the size and location of the previous frames were compared to determine whether the frame was valid. As the pupil is oval, the ellipse-fitting algorithm was applied to find a more accurate center. The performance of eye-tracking in AnyEye was evaluated by calculating the detection rate of the tracking algorithm results and compared with both CNN-based and iterative eye-tracking methods from related studies. The detection rates up to an error of five pixels of the EllSeg, PuRe, ElSe, ExCuSe were less than those of ANyEye, with detection rates of up to an error of 5 pixels of 91.26%. The detection rate of PuRe was relatively high when the error threshold was less than 1px, but ANyEye significantly outperformed other methods at a threshold above that. Analyzing the error distribution of each method in the test set, the three iterative eye tracking algorithms had a wide error distribution, while most of the results of the CNN-based algorithms had an error of less than 50 pixels. ANyEye had least standard deviation than other eye-tracking algorithms.

In this study, a slippage detection algorithm was applied to the pupil trajectory to remove slippage-induced motion artifacts in video-nystagmography. Considering the nystagmus of the lateral semicircular canal BPPV appears in the horizontal direction^41^, x-axis signal were used to determine the performance of the algorithm. Experimental results revealed that slow slippage was removed, nystagmus waveform was maintained, and fast slippage-induced motion artifacts were removed. Thus, sections likely to be confused with nystagmus were effectively removed. The section obtained through the slippage detection algorithm included motion artifact with large position changes, and the section where nystagmus occurred was not included. Compared to the slippage section marked by the expert, ANyEye selected a more delicate range, excluding the section where the slow slippage occured which could include nystagmus.

## Conclusion

In this study, we proposed ANyEye, a system including an eye-tracking algorithm and a moving-average-based slippage detection algorithm for automating BPPV diagnosis. The ANyEye outperformed both learning-based and non-learning-based algorithms. Fast slippage and slow slippage were found in the pupil trajectory data obtained from the video nystagmography dataset, and the optimal parameters for removing both types of slippages were found and applied to the slippage detection algorithm.

As this study used only one video-nystagmography device, generalization of the obtained results is limited; therefore, additional learning using the data of various devices is needed. In addition, there is a risk that using the slippage detection algorithm cannot effectively remove short-length motion artifacts compared with the parameters applied by the algorithm and distorts the nystagmus waveform over a long period. Moreover, noises generated by factors other than slipping detected in the trajectory of the pupil, such as movements of the voluntary eye or body movements of the patients during position conversion, need to be considered. To preserve the nystagmus waveform of various cycles and eliminate all types of noise, the characteristics of the nystagmus waveform and the noise appearing in the video nystagmography device need to be analyzed in more detail, and additional algorithms that adapt according to the situation need to be studied. Since our research has not been validated with external data yet, we have planned to conduct external data validation during future works. Additionally, the trajectory of the pupil of video-nystagmography videos obtained with the eye-tracking algorithm developed in this study will be used to classify the types of BPPV. Furthermore, the slippage detection algorithm is expected to minimize the errors caused by slippage during the development of diagnostic assistance algorithms.

### Supplementary Information

Supplementary Information.

## Data Availability

The datasets generated and analyzed during the current study cannot be publicly available due to patient privacy concerns but are available from the corresponding author on reasonable request.
