# Author Correction: A nystagmus extraction system using artificial intelligence for video-nystagmography

**DOI:** 10.1038/s41598-023-41913-9

**Published:** 2023-09-05

**Authors:** Yerin Lee, Sena Lee, Junghun Han, Young Joon Seo, Sejung Yang

**Affiliations:** 1 Department of Biomedical Engineering, Yonsei University, Wonju, 26493 Republic of Korea; 2 Department of Precision Medicine, Yonsei University Wonju College of Medicine, Wonju, 26426 Republic of Korea; 3 Research Institute of Hearing Enhancement, Yonsei University Wonju College of Medicine, Wonju, 26426 Republic of Korea; 4 Department of Otorhinolaryngology, Yonsei University Wonju College of Medicine, Wonju, 26426 Republic of Korea

Correction to: *Scientific Reports* https://doi.org/10.1038/s41598-023-39104-7, published online 24 July 2023

The original version of this Article contained errors in the Methods section, References 20 and 22, and the Acknowledgements section.

Supplementary Figure S1 was incorrectly cited in the Methods section, under the subheading ‘Data acquisition’. Consequently,

“The acquired data were observed to analyze slippage-induced motion artifacts, with the section where the slippage occurred indicated by two otolaryngologists (Supplementary Fig. S1).”

now reads:

“The acquired data were observed to analyze slippage-induced motion artifacts, with the section where the slippage occurred indicated by two otolaryngologists.”

The Supplementary information has now been cited in the Results section, under the subheadings ‘Segmentation performance’ and ‘Tracking evaluations’.

In addition, Reference 20 was incorrectly given as:

20. Loureiro, R. M., Sumi, D. V. & Soares, C. R. Temporal bone imaging opportunities with ultra-high-resolution computed tomography. *J. Audiol. Otol.* **27**(1), 51–53 (2023).

The correct reference is listed below:

20. Reinhardt, S., Schmidt, J., Leuschel, M., Schüle, C. & Schipper, J. VertiGo–a pilot project in nystagmus detection via webcam. *Current Directions in Biomedical Engineering* **6**(1), 20200043 (2020).

Reference 22 was incorrectly given as:

22. Jo, S., Yun, J., Kyong, J. S., Shin, Y. & Kim, J. Music Perception Abilities of the Hearing Amplification System Users. *J. Audiol. Otol.* **27**(2), 78–87 (2023).

The correct reference is listed below:

22. Keil, A., Albuquerque, G., Berger, K. & Magnor, M. A. Real-time gaze tracking with a consumer-grade video camera. In *Proceedings of the 18th International Conference in Central Europe on Computer Graphics, Visualization and Computer Vision in co-operation with EUROGRAPHICS*, 129–134 (2010).

Additionally, the Acknowledgements section contained an error.

“This work was supported by the Korean Fund for Regenerative Medicine (21A0101L0) of the National Research Foundation of Korea (Y.J.S.) and a National Research Foundation of Korea grant provided by the Korean government (Ministry of Science and ICT) (NRF-2022R1A2C2091160) (S.Y.).”

now reads:

"This research was supported by ‘Regional Innovation Strategy (RIS)’ through the National Research Foundation of Korea (NRF) funded by the Ministry of Education (MOE) (2022RIS-005) (Y.J.S.) and a National Research Foundation of Korea grant provided by the Korean government (Ministry of Science and ICT) (NRF-2022R1A2C2091160) (S.Y.)."

The original Article has been corrected.

