# Supplementary material for: A nystagmus extraction system using artificial intelligence for video-nystagmography
Source: Sci Rep. 2023 Jul 24;13:11975. doi: 10.1038/s41598-023-39104-7 (PMC10366077; doi:10.1038/s41598-023-39104-7)
Supplement: Supplementary file 1 — Supplementary Information. [file 41598_2023_39104_MOESM1_ESM.pdf]

Supplementary Materials

Figures

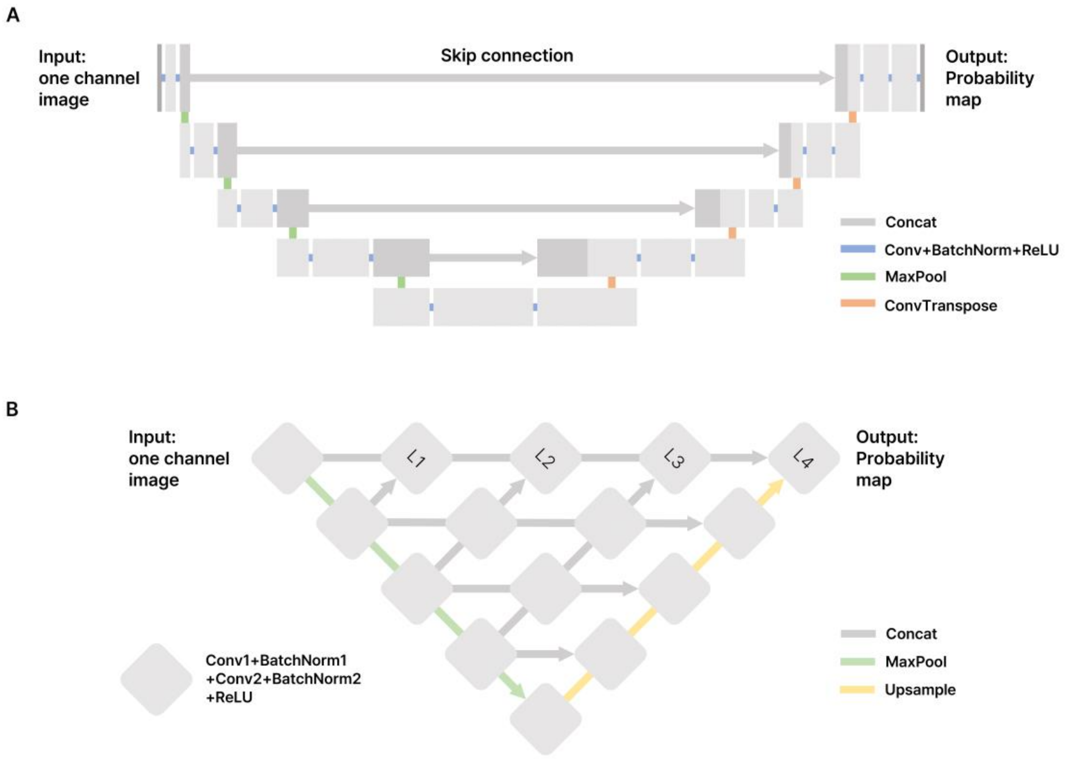

Figure S1. Architecture of CNN network used in the paper. (A) U-Net structure. (B) UNet++ structure.

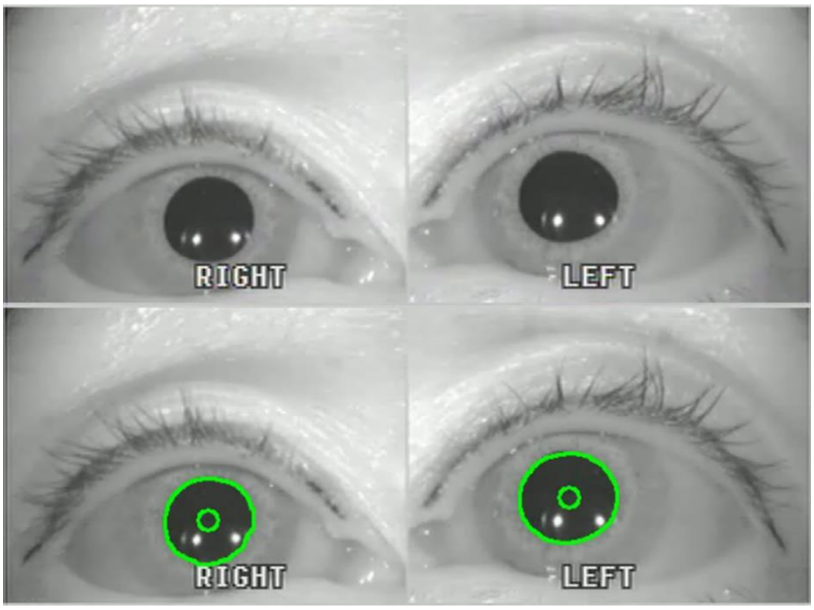

Figure S2. Sample input (first row) and output (second row) of ANyEye framework. The Python code for evaluation is provided in Github repository: https://github.com/yerinlee01/anyeye.

Tables

Table S1. Specific configurations of UNet architecture.

**Layer**	**Output**	
Contracting path		
EncoderCBR-1	64 x 128 x 128	
EncoderCBR-2	128 x 64 x 64	
EncoderCBR-3	256 x 32 x 32	[3x3, stride 1, padding 1, batchNorm, ReLU] x 2 2x2 max pool, stride 2
EncoderCBR-4	512 x 16 x 16	
EncoderCBR-5	1024 x 16 x 16	3x3, stride 1, padding 1, batchNorm, ReLU
Expanding path		
DecoderCBR-5	512 x 16 x 16	3x3, stride 1, padding 1, batchNorm, ReLU
DecoderCBR-4	256 x 32 x 32	
DecoderCBR-3	128 x 64 x 64	
DecoderCBR-2	64 x 256 x 256	2x2 conv transpose, stride 2<br>[3x3, stride 1, padding 1, batchNorm, ReLU] x 2
DecoderCBR-1	64 x 256 x 256	
FC	1 x 256 x 256	1x1, stride 1

Table S2. Specific configurations of UNet++ architecture.

Layer	Output	
Contracting path		
UnetBlock0_0	32 x 256 x 256	
UnetBlock1_0	64 x 128 x 128	
UnetBlock2_0	128 x 64 x 64	[3x3, stride 1, padding 1, batchNorm] x 2, ReLU
UnetBlock3_0	256 x 32 x 32	
UnetBlock4_0	512 x 16 x 16	
Dense skip connections		
UnetBlock0_1	32 x 256 x 256	
UnetBlock0_2	32 x 256 x 256	
UnetBlock0_3	32 x 256 x 256	
UnetBlock0_4	32 x 256 x 256	[3x3, stride 1, padding 1, batchNorm] x 2, ReLU
UnetBlock1_1	64 x 128 x 128	
UnetBlock1_2	64 x 128 x 128	
UnetBlock2_1	128 x 64 x 64	
Expanding path		
UnetBlock3_1	256 x 32 x 32	
UnetBlock2_2	128 x 64 x 64	[3x3, stride 1, padding 1, batchNorm] x 2, ReLU
UnetBlock1_3	64 x 128 x 128	
